# Learning Deep Hierarchical Spatial–Spectral Features for Hyperspectral Image Classification Based on Residual 3D-2D CNN

**DOI:** 10.3390/s19235276

**Published:** 2019-11-29

**Authors:** Fan Feng, Shuangting Wang, Chunyang Wang, Jin Zhang

**Affiliations:** 1 School of Surveying and Land Information Engineering, Henan Polytechnic University, Jiaozuo 454003, China; 211804010008@home.hpu.edu.cn (F.F.); wst@hpu.edu.cn (S.W.); 211804010001@home.hpu.edu.cn (J.Z.); 2 Center for Environmental Remote Sensing, Chiba University, Chiba 2638522, Japan

**Keywords:** hyperspectral image classification, deep learning, convolutional neural network, residual learning, depth-separable convolution, R-HybridSN

## Abstract

Every pixel in a hyperspectral image contains detailed spectral information in hundreds of narrow bands captured by hyperspectral sensors. Pixel-wise classification of a hyperspectral image is the cornerstone of various hyperspectral applications. Nowadays, deep learning models represented by the convolutional neural network (CNN) provides an ideal solution for feature extraction, and has made remarkable achievements in supervised hyperspectral classification. However, hyperspectral image annotation is time-consuming and laborious, and available training data is usually limited. Due to the “small-sample problem”, CNN-based hyperspectral classification is still challenging. Focused on the limited sample-based hyperspectral classification, we designed an 11-layer CNN model called R-HybridSN (Residual-HybridSN) from the perspective of network optimization. With an organic combination of 3D-2D-CNN, residual learning, and depth-separable convolutions, R-HybridSN can better learn deep hierarchical spatial–spectral features with very few training data. The performance of R-HybridSN is evaluated over three public available hyperspectral datasets on different amounts of training samples. Using only 5%, 1%, and 1% labeled data for training in Indian Pines, Salinas, and University of Pavia, respectively, the classification accuracy of R-HybridSN is 96.46%, 98.25%, 96.59%, respectively, which is far better than the contrast models.

## 1. Introduction

Hyperspectral sensors can collect abundant spectral information of target objects in hundreds of narrow bands. Hyperspectral images have extremely high spectral resolution, pixel-wise classification of which is the cornerstone of various hyperspectral applications, including agricultural yield estimation [1], environment monitoring [2], resource surveying [3], and disaster monitoring [4]. However, hyperspectral classification is still challenging due to high dimensionality, high nonlinearity, and the “small-sample problem” of hyperspectral data [5,6].

Research of hyperspectral classification is focused on the feature extraction and classifier designing [7]. Classic feature extraction methods include principal components analysis (PCA) [8], independent components analysis (ICA) [9], linear discriminant analysis [10], etc., aiming at dimensionality reduction and feature discrimination enhancement of hyperspectral data. Subspace clustering, which has a good theoretical foundation, is an important unsupervised representation learning method for high dimensional and large-scale data analysis [11,12]. Recently, subspace clustering has been successfully applied to hyperspectral classification in both an unsupervised manner and a supervised manner [13,14]. It is noteworthy that subspace clustering can be used as a powerful tool for band selection [15,16]. Classifiers designing combined with pattern recognition and machine learning is an effective solution for hyperspectral classification, and representative algorithms include support vector machine (SVM) [17], multinomial logistic regression (MLR) [18], extreme learning machines (ELMs) [19], etc. Most earlier studies are based on spectral information, while spatial information has attracted more attention recently. Furthermore, a large number of literatures indicate that classification utilizing spatial–spectral information can effectively remove salt–pepper noise and generate better predicted maps [20,21,22,23]. High dimensionality, high nonlinearity, and limited training samples of hyperspectral data require feature extraction methods and classifiers to be capable of extracting and processing deep abstract features, making feature extraction the core issue of hyperspectral classification [24]. However, most above-mentioned learning methods and classifiers, such as PCA, ICA, MLR, SVM, etc., are not based on a “deep” manner and deep architectures; on the contrary, can promote the re-use of features and learn more abstract features at higher layers of representations [25]. For example, subspace clustering when employed as a deep model can better handle realistic data without the linear subspace structure [26]. In recent years, deep learning has attracted much attention by providing an ideal solution for feature extraction. On the one hand, a deep learning model can extract features through active learning, with less human intervention and strong generalization ability [27]. On the other hand, deep structures can extract hierarchical features with increasing levels of abstraction [28]. Different from deep learning models, which take 1D vector as input, such as the stacked auto-encoder (SAE) [29] and deep belief network (DBN) [30], the convolutional neural network (CNN) [31] can directly process 2D or 3D image data. Many important breakthroughs have been made in the field of computer vision by applying CNN, such as image classification [31,32,33,34], object detection [35], and semantic segmentation [36]. A large number of literatures have shown that CNN can effectively extract the deep spatial–spectral features directly from raw hyperspectral data blocks and achieve ideal classification accuracy [37,38,39,40,41,42].

A deep CNN model called AlexNet won the championship of the ImageNet Large Scale Visual Recognition Challenge (ILCVRS) in 2012, creating a giant wave of solving image processing issues with the study of the CNN model. Since then, VGG [32], GoogleNet [33], ResNet [34], and other networks with excellent performance in the ILCVRS competition have overcome one milestone after another in CNN model research. In addition, the lightweight design of the CNN model has attracted more attention in recent years, aiming at reducing computational complexity. Xception [43], SqueezeNet [44], MobileNet [45], ShuffleNet [46], and other lightweight CNN models have been proposed successively. Researches on the CNN model strongly promote its penetration into related image processing areas. CNN-based hyperspectral classification mainly focus on hierarchical feature extraction with 2D-CNN or 3D-CNN. 2D-CNN-based hyperspectral classification mostly extract spatial features form hyperspectral data after dimension reduction [37,38]. The number of retained spectral bands and spatial window size vary from model to model. However, because of spectral information loss during 2D convolutional operation, the accuracy of 2D-CNN-based hyperspectral classification can be improved by utilizing 3D-CNN to extract spatial–spectral features. Chen et al. extract the deep spatial–spectral features from hyperspectral data using the 3D convolution kernel [39]. The spatial window size is 27 × 27 and kernel size is 5 × 5 × 32. Li et al. built a 3D-CNN-based model with relatively small kernel and window size [40]. Moreover, features with stronger discrimination can be extracted by optimizing the network structure of 3D-CNN. Hamida et al. alternatively used 3D convolution kernel of different scales to extract spatial–spectral features [41]. He et al. used multi-scale convolution kernel in the same convolutional layer to extract spatial–spectral features, and then concatenated the extracted spatial–spectral features for hyperspectral classification [42].

Hyperspectral data annotation is time-consuming and laborious, resulting in very limited labeled samples which can be used for training models. The “small-sample problem” has attracted more and more attention recently [47,48]. There are several mainstream methods in the machine learning field to solve the “small-sample problem” of hyperspectral classification, which are data augmentation [39,49], semi-supervised learning [50,51,52], transfer learning [53,54], and network optimization [55,56,57,58]. Unlike the other three strategies, network optimization focuses on the model itself. Based on above observations, further optimizing the network structure to extract more discriminative features, and meanwhile improving the parameter using efficiency to avoid overfitting, is the focus of hyperspectral classification research based on 3D-CNN.

In terms of extracting discriminative features, theoretical research and image processing practice have proved that network depth is the key factor [32,33,34,59,60]. By using residual connections in ResNet [34], some researchers construct relatively deeper networks for hyperspectral classification. Lee et al. use 1 × 1 convolution kernels to learning hierarchical features extracted by the previous multi-scale convolution kernels, in which residual connections are used to make a network deeper [55]. Liu et al. built a Res-3D-CNN of 12 layers with 3D convolution kernels and residual connections [56]. Zhong et al. improved the position of residual connections and built a SSRN (Spectral–Spatial Residual Network) in which residual connections are used in the spectral feature learning and the spatial feature learning, respectively [57]. In addition, structure innovation is also an important aspect of network optimization. Lately, Swalpa K R et al. proposed HybridSN, applying a 2D convolutional layer to further processing spatial–spectral features extracted by successive 3D convolutional layers [58]. This concise and elegant model shows the giant potential of 2D-3D-CNN in hyperspectral classification by comparing it with previous state-of-the-art models, such as SSRN.

In order to better solve the “small-sample problem” of hyperspectral classification, we proposed a deeper 2D-3D-CNN called R-HybridSN (Residual-HybridSN). R-HybridSN not only inherits the advantages of some existing models, but also has considerably innovative designs. With a deep and efficient network structure, our proposed model can better learn deep hierarchical spatial–spectral features. Specially, the depth-separable convolutional layer proposed in Xception [43] is used to replace the traditional 2D convolutional layer, aiming to reduce the number of parameters and further avoid overfitting.

## 2. Methodology

### 2.1. Proposed Model

**Figure 1:**
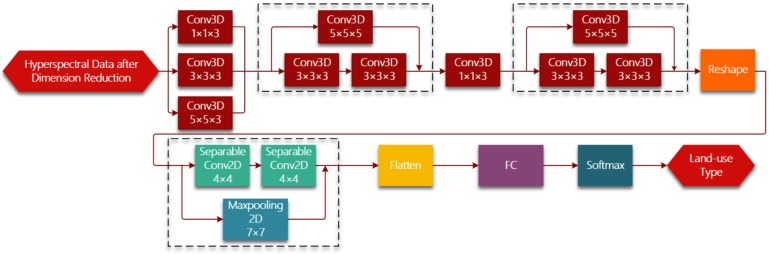
Illustration of the proposed R-HybridSN (Where FC is the fully connected layer).

Figure 1 shows the whole framework of hyperspectral imagery classification based on R-HybridSN. In the light of lightweight designing, we conduct dimension reduction on raw hyperspectral data using PCA and keep a relatively small number of principle components. Hyperspectral data can be viewed as a 3D cube. Suppose the spatial size of hyperspectral data is W×H and the number of spectral bands is D, then the hyperspectral 3D cube can be denoted as $C_{W\times H\times D}\;$ PCA is used in the spectral dimension of hyperspectral data. After selecting the first B principle components, the hyperspectral data can be denoted as $C_{W\times H\times B}\;$. The proposed R-HybridSN is based on 3D-CNN and accepts the 3D hyperspectral image patch as input, the land-use type of which is determined by the center pixel. A hyperspectral patch includes not only the spectral information of the pixel to be classified, but also the spatial information of the pixel within a certain range around. Every hyperspectral patch can be denoted as $P_{M\times M\times B}$, where M is the predefined neighborhood size.

The R-HybridSN has 11 layers and Table 1 shows the output dimension of each layer, the number and size of convolution kernels. Reshape and Flatten module is applied for data dimension adjustment, the purpose of which is adapt to the data dimension requirement of next layer. Deep hierarchical spatial–spectral features are extracted by six successive 3D convolutional layers and two depth-separable 2D convolutional layers. In addition, we chose the widely used rectified linear unit (Relu) as the nonlinear activation function. After further abstract integration of spatial–spectral features, every pixel is classified with a specific land-use type in the output layer. Three residual connections are introduced to ease the training process of R-HybridSN and information from the upstream of the network is injected downstream. Specifically, the residual connections used in R-HybridSN include dimension adjustment. The position and dimension adjustment methods are shown in the dotted box in Figure 1.

### 2.2. The 2D Convolution Operation

**Figure 2:**
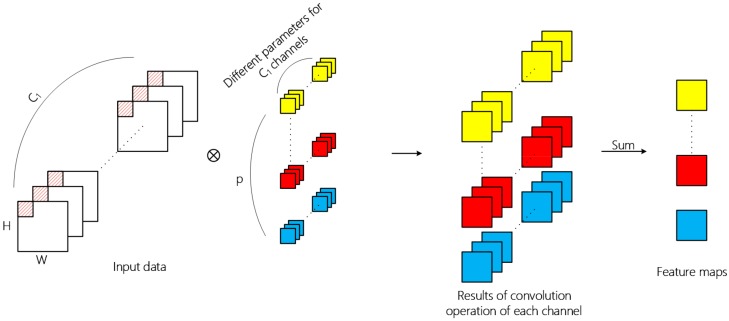
The overall 2D convolution operation. The input data dimension is $W\times H\times C_{1}\;\;$, where $C_{1}$ is the channel number; the 2D convolution kernel size is k×k and it denotes the coverage of convolution kernel over spatial dimension in each convolution operation; the output data dimension of single kernel is $\left(W-k+1\right)\left(H-k+1\right)$ and the final output data generated by p kernels is a 3D tensor.

Convolutional neural networks are usually composed of input layer, convolutional layer, pooling layer, fully connected layer, and output layer. The convolutional layer is the core component for the extraction of deep hierarchical features. A convolutional layer extracts features by means of inner product of kernels in this layer and input data of the previous layer, in which convolutional kernel traverses all spatial positions of each channel of input data. The overall 2D convolution operation is shown in Figure 2 and the value of the $\left(x,\;y\right)$ position on the jth feature map at the ith layer is calculated by Formula (1),

(1) $$map_{i,j}^{x,y}=f\left(\sum_{m}\sum_{h=0}^{H_{i}-1}\sum_{w=0}^{W_{i}-1}k_{i,j,m}^{h,w}map_{\left(i-1\right),m}^{\left(x+h\right),\left(y+w\right)}+b_{i,j}\right),$$

where $k_{i,j,m}^{h,w}$ represents the value at the position $\left(h,\;w\right)$ of the jth convolution kernel in the ith layer, and the kernel convolutes the m th feature map of the previous layer; $H_{i}$ and $W_{i}$ represnets the size of the kernel; $map_{i-1,m}^{\left(x+h\right),\left(y+w\right)}$ represents the value at the position $\left(x+h,\;y+w\right)$ of the mth feature map of the previous layer; $map_{i,j}^{x,y}$ represents the output at the position $\left(x,\;y\right)$ of the jth feature map in the ith layer; $b_{i,j}$ represents the bias and f() is the activation function. Different parameters are used for convolution operation in different channels, and the two-dimensional tensor obtained by summation of operation results is the feature extracted by the convolution kernel.

Through 2D convolution operation, one layer can learn local features from the previous layer and the kernel size determines local spatial size. Successive convolutional layer can increasingly abstract hierarchical features. In addition, the spatial feature learning and cross-channel feature learning in the ordinary 2D convolutional layer is carried out simultaneously. For the input 3D image data with length, width, and band, the operation result of multiple convolution kernels is also a 3D tensor. However, the depth dimension no longer corresponds to the band, but to the number of convolution kernels. 

### 2.3. The 3D-CNN and 2D-3D-CNN

**Figure 3:**
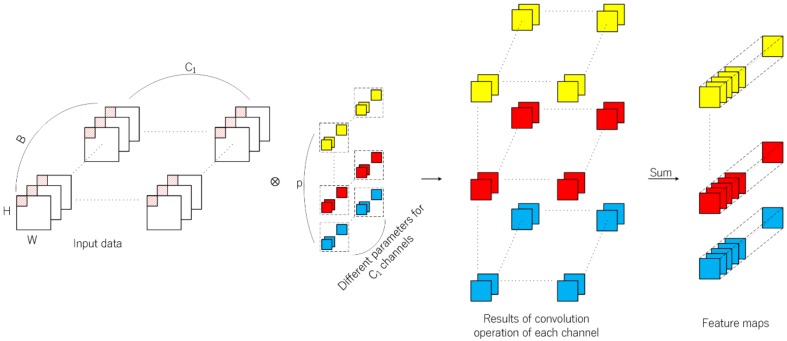
The overall 3D convolution operation. The input data dimension is $W\times H\times B\times C_{1}\;\;$, where B is the band number and $C_{1}$ is the channel number; the 3D convolution kernel size is k×k×k and the last k denotes the coverage of convolution kernel over spectral dimension in each convolution operation; if padding is not used and the stride is 1, then the output data dimension of single kernel is $\left(W-k+1\right)\left(H-k+1\right)\left(B-k+1\right)$ and the final output data generated by p kernels is a 4D tensor.

Different from 2D-CNN, feature learning is extended to the depth dimension by using 3D convolution kernels. The 3D convolution kernels conduct inner product across the spatial dimension and the depth dimension. Thus, it is suitable for the feature learning of hyperspectral data with rich spectral information. Firstly, R-HybridSN extracts spatial–spectral features using multi-scale 3D convolution kernels, in which padding is used to ensure the consistent dimension of input and output data. The operation results are concatenated in the spectral dimension as the output feature map of the first layer. The next step is to further integrate and abstract the spatial–spectral features with 5 successive convolutional layers. The process of 3D convolution operation is shown in Figure 3. The calculation method of the position $\left(x,\;y,\;z\right)$ of the jth feature cube in the ith convolutional layer is shown in Formula (2).

(2) $$V_{i,j}^{x,y,z}=f\left(\sum_{m}\sum_{h=0}^{H_{i}-1}\sum_{w=0}^{W_{i}-1}\sum_{c=0}^{C_{i}-1}k_{i,j,m}^{h,w,c}V_{\left(i-1\right),m}^{\left(x+h\right),\left(y+w\right),\left(z+c\right)}+b_{i,j}\right),$$

3D Convolution and 2D convolution have their own characteristics in feature extraction. In the 3D-CNN-based hyperspectral classification task, let the input data dimension, convolution kernel size, and the kernel number be $W\times H\times B\times C_{1},k\times k\times k,p,$ respectively. In the 2D-CNN-based hyperspectral classification task, let the input data dimension, convolution kernel size, and the kernel number be $W\times H\times C_{2},k\times k,p\;\;$, respectively. If padding is not used and the stride is 1, then the dimension of feature map is $\left(W-k+1\right)\left(H-k+1\right)\left(B-k+1\right)p$ generated by 3D convolutional layer, and $\left(W-k+1\right)\left(H-k+1\right)p$ generated by 2D convolutional layer, respectively. In terms of network parameters, the number of weight parameters is $p\times k\times k\times k\times C_{1}$ for the 3D convolutional layer, and $p\times k\times k\times C_{2}$ for the 2D convolutional layer. Therefore, on the one hand, the feature map generated by the 3D convolution operation contains more spectral information. On the other hand, the parameters of 3D convolutional layer are usually far more than those of 2D convolutional layer.

Inspired by HybridSN, the 2D convolutional layer is placed behind the successive 3D convolutional layers to further discriminates the spatial features. In order to fit the input dimension of 2D convolutional layer, the feature cubes generated by the 3D convolutional layer should be jointed in the spectral dimension, namely the dimension of the 4D tensor W×H×B×C should be reshaped to W×H×BC  . Different from HybridSN, R-HybridSN utilizes two depth-separable convolutional layers to enhance parameters using efficiency and residual connections to ease network training and to enhance the flow of spectral information in the network.

### 2.4. Depth-Separable Convolution

**Figure 4:**
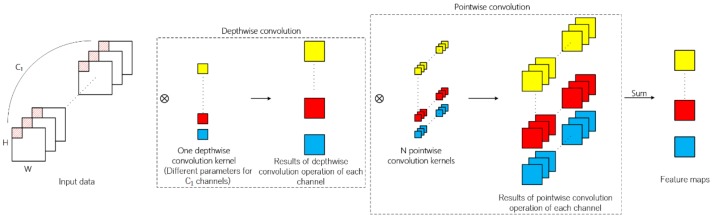
The overall depth-separable convolution operation. Different from traditional 2D convolution, depth-separable convolution can be divided to depthwise convolution and pointwise convolution.

It can be observed from the operation process of traditional 2D convolution that in terms of feature map generation, the information of spatial dimension and channel dimension is mapped simultaneously. Different from traditional 2D convolution, depth-separable convolution can be divided into depthwise convolution and pointwise convolution. Firstly, every channel of input data is convoluted by 2D convolution kernels and the number of kernels is usually set to one in depthwise convolution. The second step is similar to traditional 2D convolution of which kernel size is 1 × 1. The above operation process is illustrated in Figure 4.

Compared with the traditional 2D convolution, depth-separable convolution both reduces the number of parameters and the calculation times in the network, thus speeding up the network training and reducing the possibility of overfitting in the classification task. Suppose that the channel number of input feature map is C, the size of convolution kernel is K×K  , the number of depthwise convolution kernel is *q*, the number of pointwise convolution kernel is *N*, and the size of the generated feature map is W×H×N  . The number of parameters in the traditional convolutional layer is K×K×N×C and the number of parameters in the depth-separable convolutional layer is N×C×q+K×K×C×q  . The times of multiplications is K×K×C×W×H×N for the traditional convolutional layer and 1×1×W×H×N×C×q+W×H×K×K×C×q for the depth-separable convolutional layer, respectively. The ratios on parameters and times of multiplications of traditional convolutional layer to depth-separable convolutional layer are both $\left(\frac{q}{N}+\frac{q}{K^{2}}\right)$. Since *q* is usually set to 1, using depth-separable convolution can greatly reduce the number of parameters and the calculation times required.

The performance of hyperspectral classification can be improved by applying depth-separable convolution. First, the number of parameters and calculation times are reduced. Second, the concatenated feature map generated by successive 3D convolutional layers can be considered to contain extremely abundant spectral information in a certain neighborhood of the pixel to be classified. The depth-separable convolution is very suitable for the data structure of the concatenated feature map, of which the size depth dimension is far larger than that of spatial dimension.

### 2.5. Residual Learning

The theoretical research on the feedforward deep network indicates that the depth of the network has an exponential advantage over the width of the network (namely the number of neurons in the same layer) in terms of function fitting ability [59,60]. In addition, the image classification practice based on CNN shows that the deep network can learn abstract features better [32,33,34]. Therefore, deep network construction is an important strategy to deal with the challenge of hyperspectral classification. And in terms of constructing deep network for “small-sample problem”, the key point is reducing training difficulty and avoid overfitting.

**Figure 5:**
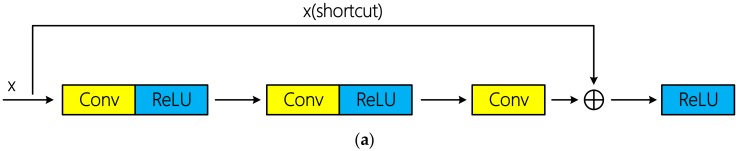

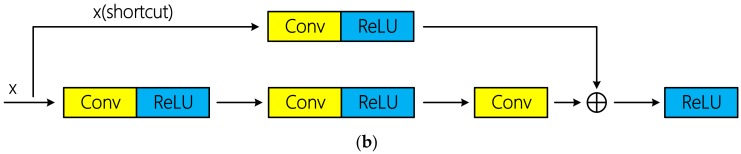
Schematic diagrams of two types of residual connections: (**a**) Identity connection; (**b**) non-identity connection using convolutional layer for dimension adjustment.

The training of the deep convolutional neural network can be successfully realized through residual connection proposed in literature [34]. Residual connections can be divided into identity connection and non-identity connection according to whether there is dimension adjustment. Schematic diagram of the two types of residual connections is shown in Figure 5. Tensor addition requires same dimension. If the input x does not change its dimension after several convolutional layers, x can be directly injected into the downstream of the network by using identity connection; otherwise, dimension adjustment is required. Specially, [34] indicated that identity connection is sufficient to solve the degradation problem of deep network, and is more economical and practical than non-identity connection with dimension adjustment. In the mentioned hyperspectral classification researches above based on residual learning, all the authors used identical connection and achieved excellent classification performance.

However, there are three non-identity residual connections in R-HybridSN. The first two residual connections occurred among the successive 3D convolutional layers, and the 3D convolutional layer was used to conduct the dimension adjustment. In addition, the dimension adjustment in the third residual connection is done by a max pooling layer. The reasons why identical residuals are not used in this article are as follows:

1. From the perspective of network structure, identical connection requires consistent input and output dimensions, which reduces the flexibility of model construction to some extent. Thus, we aim to explore the classification performance of hyperspectral data using non-identity residual connection.
2. Residual connections with convolutional layers make the network more like directed acyclic graphs of layers [61], in which each branch has the ability to independently extract spectral-spectral features.
3. The feature map generated by the Reshape module is believed to contain extremely rich spectral information. However, due to the mode of feature map generating used by subsequent 2D convolutional layers, the extracted spectral features suffer some losses. In order to better realize spatial–spectral association, we add the feature map generated by the Reshape module to the feature map generated by the last 2D convolutional layer, enhancing the flow of spectral information in the R-HybridSN.

## 3. Datasets and Contrast Models

To verify the performance of the proposed model, we use three public available hyperspectral datasets, namely Indian Pines, Salinas, and University of Pavia. The Indian Pines, Salinas, and University of Pavia datasets are available online at http://www.ehu.eus/ccwintco/index.php?title=Hyperspectral_Remote_Sensing_Scenes. We use some public available codes to prepare the training and testing data, compute the experimental results and implement the M3D-DCNN as one of the contrast models. The codes are available online at https://github.com/gokriznastic/HybridSN and https://github.com/eecn/Hyperspectral-Classification. Indian Pines consists of 145 × 145 pixels with a spatial resolution of 20 m. The bands covering the water absorption region were removed and the remaining 200 bands are used for classification. The Indian Pines scene mainly contains different types of crops, forests, and other perennials. The ground truth available is designed to sixteen classes. Salinas consists of 512 × 217 pixels with a spatial resolution of 3.7 m. As with Indian Pines, the water absorption bands were discarded and the number of remaining bands is 204. The Salinas scene mainly consists of vegetation, bare soil, and vineyards. The labeled samples are divided into 16 classes, total of which is 54,129. The University of Pavia consists of 610 × 340 pixels with a spatial resolution of 1.3 m. The labeled samples are divided into 9 classes, most of which are the features of the town, such as metal plate, roof, asphalt pavement, etc. The total number of labeled samples is 42,776.

Most of CNN-based hyperspectral classification algorithms are supervised and the number of training samples are of great significance to the classification accuracy. The same three datasets were used in [58] and the proportion of training samples used was 30%. In addition, supplementary experiments using 10% labeled data as training samples were conducted to further observe the performance of HybridSN. In [57], 20% labeled data were used to train SSRN for Indian Pines, and 10% for University of Pavia, respectively.

In our experiment, the number of training samples was further reduced to observe the classification performance of R-HybridSN. In addition, different from the common setting of most hyperspectral classification experiments, we do not use the same amount of training samples for each class in this paper. Instead, the training sample ratio of each ground object class is consistent with that in the total labeled samples. Three landmark CNN-based hyperspectral classification models—2D-CNN [37], M3D-DCNN [42], and HybridSN [58]—are compared with our proposed R-HybridSN. In addition, in order to observe the impact of residual learning and depth-separable convolution in R-HybridSN on classification performance, we built the following two extra contrast models, namely Model A and Model B. Model A replaces the part of depth-separable convolution with traditional 2D convolution and Model B removes residual connections from R-HybridSN. The other settings are consistent with R-HybridSN. 

## 4. Experimental Results and Discussion

Details of 2D-CNN, M3D-DCNN, and HybridSN are set in accordance with the corresponding paper. In order to better investigate the training speed, the training epoch of R-HybridSN, Mode A, and Model B is set to 50. We use Adam as optimizer, and the learning rate is set to 0.001. In addition, in order to observe the impact of model structure itself on training speed, normalization strategies, such as batch normalization [62], are not used.

### 4.1. Hyperspectral Classification Experiments Using Different Amounts of Training Samples

In this section, multiple hyperspectral classification experiments were conducted with different amounts of training samples, and the classification performance of different models was evaluated by overall accuracy (OA). The OA is calculated by the number of correctly classified testing pixels divided by the total number of testing pixels. The purpose of these experiments is to observe the variation trend and sensitivity of OA with the changing amount of training samples. In particular, in this section, we remove seven classes with too few labeled samples in Indian Pines referring to literature [56]. If not, under the rigorous experimental settings, some ground object may not have even one training sample. The OA of each model on Indian Pines, Salinas, and University of Pavia is shown in Table 2, Table 3 and Table 4. In addition, the training time of each model on Indian Pines is shown in Table 5. The data in the table were obtained by averaging the results of ten consecutive experiments.

As can be seen from the classification accuracy results, when the training samples accounted for 20% of the total number of labeled samples, all models reached the highest accuracy. 2D-CNN and M3D-DCNN have lower accuracy than other models. The classification accuracy of all models decreased with the decrease of training samples and the reduction speed of different models varies greatly. In all experiments in this section, HybridSN has higher classification accuracy than 2D-CNN and M3D-DCNN. Combined with the experimental conclusions in [58], the experimental results in this section further show that the combination of 2D and 3D convolutional layers can effectively improve the accuracy of hyperspectral classification. The classification accuracy of R-HybridSN in Indian Pines and University of Pavia is far better than all other models, and the advantages of R-HybridSN gradually increase as the amount of training samples decreases. For example, in Indian Pines_10%, the classification accuracy of R-HybridSN is 0.78% higher than HybridSN. Furthermore, as the proportion of training samples decreases from 10% to 2%, the classification accuracy of R-HybridSN is successively 1.75%, 3.05%, 3.11%, and 3.53% higher than HybridSN. This trend is even more pronounced in University of Pavia. In the Salinas data set, the classification accuracy of R-HybridSN is slightly lower than HybridSN and superior to all the other models. Specially, the gap between R-HybridSN and HybridSN in Salinas does not expand as the amount of training samples decreases. In the process of reducing the proportion of training samples from 5% to 2%, the classification accuracy of R-HybridSN was at most 0.81% lower than HybridSN, which occurred in the Salinas_0.8% experiment, while in Salinas_0.4%, the gap was reduced to 0.55%. We can tell by the experimental results that the network structure of R-HybridSN is good enough to extract sufficient discriminative features using a very few training samples, so as to maintain a high classification accuracy. The reason why the accuracy of R-HybridSN is slightly lower than HybridSN in Salinas will be further discussed in Section 4.2.

The classification accuracy of Model A is higher than Model B, and that of Model A and Model B is far lower than R-HybridSN. Again, this trend became more pronounced as the number of training samples decreased. For example, the classification accuracy of R-HybridSN is 1.5% higher than Model A in the Indian Pines_10% experiment, and as the proportion of training samples decreases from 10% to 2%, the classification accuracy of R-HybridSN is successively 1.09%, 2.89%, 3.79%, and 4.7% higher than Model A. Therefore, depth-separable convolution and residual connections used in R-HybridSN can effectively improve the classification accuracy. In Salinas_0.4% and University of Pavia_0.4%, the classification accuracy of Model B is even lower than that of 2D-CNN, indicating that serious degradation exists in the deep CNN model. In particular, it is noted that in University of Pavia, when the proportion of training samples decreased from 1.2% to 0.8%, the classification accuracy of Model B increased from 88.54% to 91.05% on the contrary. It can be observed from the standard deviation that the classification accuracy of model B on the University of Pavia_1.2% is very unstable. This phenomenon may be caused by the fact that the model without residual connections was more difficult to train, and the training accuracy can hardly be improved to 100% within 50 epochs. Once this situation occurs, there will be a great impact on the calculated average accuracy. Detailed observation and further analysis of this phenomenon is shown in Section 4.2.

The training time of deep learning model is related to experimental environment, model structure, number of training epoch, amount of training samples, etc. The training time of each model varies greatly and all decrease with the decreasing amount of training samples. 2D-CNN has the shortest training time, while the training time of 3D-CNN and HybridSN are relatively long. The training time of R-HybridSN is moderate, higher than 2D-CNN, lower than M3D-DCNN and HybridSN. The training epoch of the last three models in Table 5 are all 50. The training time of R-HybridSN is shorter than that of Model A, and longer than that of Model B. The experimental results indicate that the depth-separable convolution can not only improve the classification accuracy, but also shorten the training time, while the introduction of non-identical residual connection can prolong the training time.

### 4.2. Hyperspectral Classification Experiments under “Small-Sample Problem”

The problems exposed in the previous section should be further observed and the “small-sample problem” will be paid more attention. Therefore, in this section, a small sample proportion was fixed to further observe the classification effect of each model from various indices. In this section, the proportion of training samples used in Indian Pines, Salinas and University of Pavia is 5%, 1%, and 1%, and the proportion of training samples selected for each class of ground object is consistent with the proportion of such ground object in the total number of labeled samples. Different from the experiments in Section 4.1, all the 16 classes in Indian Pines are included in this section. In light of the distribution of training samples are highly unbalanced and some ground object have only one or two training samples, the IP_5% experiment in this section can be viewed as one extreme condition of “small sample” hyperspectral classification. The detailed distribution of training samples and test samples of Indian Pines, Salinas and University of Pavia are shown in Table 6, Table 7 and Table 8.

In this section, three indices were used to evaluate the classification performance of different models, namely OA, AA, Kappa. AA refers to the average accuracy of all ground features, and it is calculated by the arithmetic average of the classification accuracy of all ground objects. Kappa is an index measuring classification accuracy based on confusion matrix, and its calculation method is shown in Equation (3), where $t_{i}$ is the true sample number of the ith class sample, $p_{i}$ is the predicted number of *i*th class sample, n is the total number of sample, and k is the number of sample classes.

(3) $$Kappa=\frac{OA-\frac{t_{1}p_{1}+t_{2}p_{2}+\ldots +t_{k}p_{k}}{n^{2}}}{1-\frac{t_{1}p_{1}+t_{2}p_{2}+\ldots +t_{k}p_{k}}{n^{2}}},$$

**Figure 6:**
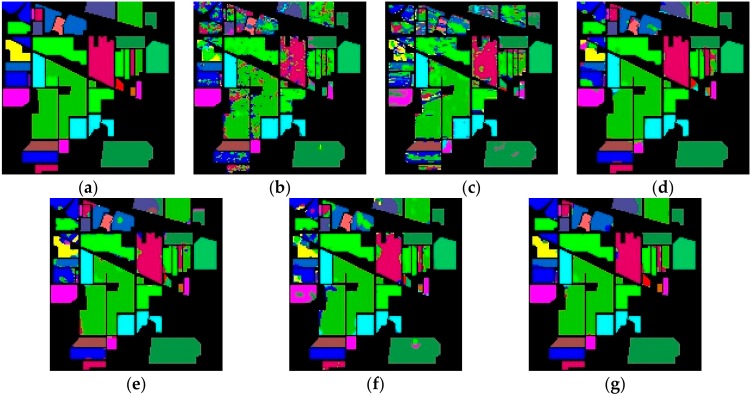
The classification maps of Indian Pines. (**a**) Ground truth. (**b**–**g**) Predicted classification maps for 2D-CNN, M3D-CNN, HybridSN, Model A, Model B, and R-HybridSN, respectively.

**Figure 7:**
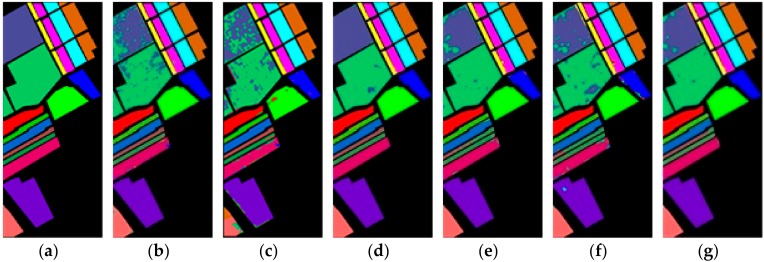
The classification maps of Salinas. (**a**) Ground truth. (**b**–**g**) Predicted classification maps for 2D-CNN, M3D-CNN, HybridSN, Model A, Model B, and R-HybridSN, respectively.

**Figure 8:**
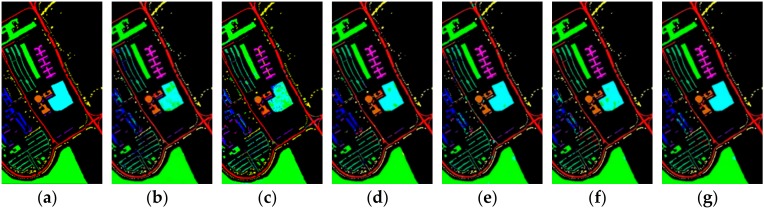
The classification maps of University of Pavia. (**a**) Ground truth. (**b**–**g**) Predicted classification maps for 2D-CNN, M3D-CNN, HybridSN, Model A, Model B, and R-HybridSN, respectively.

Figure 6, Figure 7 and Figure 8 show the ground truth of Indian Pines, University of Pavia, and Salinas and the classification map generated by different models in one certain experiment. As can be seen from these figures, in this experiment, the classification effect of R-HybridSN and HybridSN is far better than that of 2D-CNN and M3D-CNN. In addition, the classification effect of R-HybridSN is better than that of Model A and Model B. Table 9 shows the classification result of the most seriously misclassified ground object by R-HybridSN in Salinas, which will be discussed in detail later. Table 10, Table 11 and Table 12, respectively, show the test results of R-HybridSN and five contrast models in Indian Pines, Salinas, and University of Pavia. The data in these tables were obtained by averaging the results of ten consecutive experiments. Similar to the experimental results in Section 4.1, R-HybridSN are superior to all the remaining models in Indian Pines and University of Pavia. For example, in Indian Pines, the OA of R-HybridSN is 20.99%, 27.58%, 2.22%, 3.49%, and 6.66% higher than 2D-CNN, M3D-DCNN, HybridSN, Model A, and Model B, respectively. In the University of Pavia, R-HybridSN has an advantage of 1.5% over the suboptimal Model HybridSN in terms of OA and 3.25% in terms of AA. In Salinas, the Kappa, OA, AA of R-HybridSN are only slightly lower than HybridSN.

The experimental results in Table 11 indicate that the reason that the classification effect of R-HybridSN is slightly lower than that of HybridSN in the Salinas is that Lettuce_romaine_6wk, the thirteenth class of ground object, has a poor classification result. Table 9 shows the confusion of Lettuce_romaine_6wk in the 10 experiments of R-HybridSN in Salinas. Lettuce_romaine_6wk is mainly misclassified as Lettuce_romaine_7wk and Lettuce_romaine_5wk. Meanwhile, the classification effect of Model A and Model B on Lettuce_romaine_6wk is lower than that of R-HybridSN, while the classification effect of 2D-CNN, M3D-DCNN, and HybridSN on Lettuce_romaine_6wk is much better than that of R-HybridSN. Based on this phenomenon, it is speculated that the specific network structure of R-HybridSN may be the reason that the classification effect of thirteenth class of ground object is unsatisfactory.

**Figure 9:**
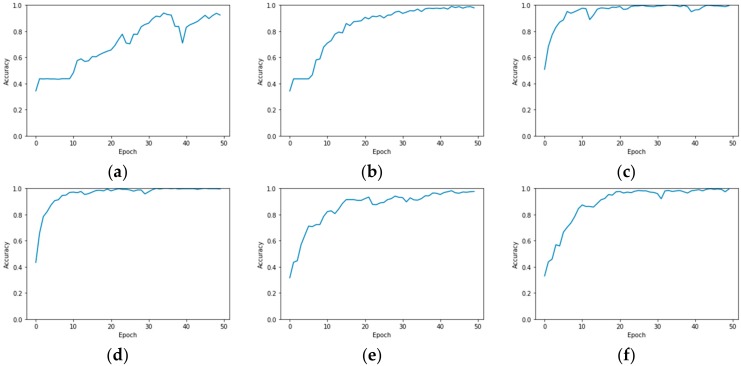
Training accuracy curves in six consecutive experiments (**a**) Model B_1.2%, OA = 93.37%; (**b**) Model B_1%, OA = 94.01%; (**c**) R-HybridSN_1.2%, OA = 97.52%; (**d**) R-HybridSN_1%, OA = 97.03%; (**e**) Model A_1.2%, OA = 95.95%; (**f**) Model A_1% OA = 96.39%.

It is noted that the classification accuracy of Model B on the University of Pavia using 1% labeled samples for training is higher than that of using 1.2% labeled samples for training, with a difference of 5.96%. This is inconsistent with the overall experimental phenomenon that the classification accuracy decreases with the decrease of the amount of training samples. Figure 9 shows the changing curves of the training accuracy with the training epoch of R-HybridSN, Model A, and Model B using 1% and 1.2% labeled samples for training, respectively. These curves were obtained in six consecutive experiments. It can be seen from the figure that Model B could get fully trained (the final training accuracy was close to 1) within 50 epochs using 1% labeled samples. The reason for this phenomenon may be that 1% samples had a special coupling relationship with the batch size that we set, which could make the training smooth. In addition, it can be seen that R-HybridSN and Model A could be fully trained within 50 epochs under the same condition of batch size and training samples. In particular, the training accuracy of R-HybridSN increases most rapidly, and it could reach nearly 100% within about 20 epochs. The significance of residual connection for the training of deep CNN is further verified.

### 4.3. Discussion

The CNN-based hyperspectral classification model degenerates with the decrease of the amount of training samples. Roughly, the models with fewer layers and simpler structure has more serious degeneration problem. The good performance of HybridSN with limited training samples shows the importance of network optimization and the potential of 2D-3D-CNN in hyperspectral classification. The performance of proposed R-HybridSN is verified by a series of rigorous experiments and its classification effect is far better than HybridSN and other contrast models in Indian Pines, University of Pavia and slightly lower than HybridSN in Salinas. The significance of residual connections and depth-separable convolution layers has already been demonstrated. Next, the structural advantage of R-HybridSN will be further discussed.

Constructed on the idea of network optimization, R-HybridSN is designed to extract robust and discriminative features while mitigating over-fitting. Compared with the most existing CNN-based hyperspectral classification models, R-HybridSN is more like directed acyclic graphs of layers. This structural advantage of R-HybridSN might provide a better solution for “small sample” hyperspectral classification. On the one hand, R-HybridSN has more ways for feature extraction besides the main path. For example, the multiscale convolution module can exploit spatial structure as well as spectral correlations [55]. Every non-identity residual connection can learn spatial–spectral features independently. On the other hand, convolutional layers in R-HybridSN is rich in variation in terms of category and kernel size, and they are arranged in a carefully designed order which can better control over-fitting. For example, different 3D convolutional layers are combined with depth-separable convolutional layers, providing a novel hierarchical spatial–spectral learning pattern. The tail branch of R-HybridSN, namely the max pooling layer, should be paid more attention. We remove the third residual connection in R-HybridSN and supplementary experiments have been conducted. The comparison between whether the third residual connection is used in R-HybridSN is shown in Table 13 and the structural advantage of R-HybridSN is further demonstrated. In the last few layers, the number of convolution kernels increased sharply, meanwhile the spatial size of the output feature map was reduced to the minimum (1 × 1). While increasing the feature discrimination degree, overfitting has been effectively controlled. Based on the above settings, the pooling layer adds the feature map generated by the last 3D convolutional layer to the feature map generated by the last depth-separable convolutional layer, boosting the flow of spectral features in the whole network. To sum up, the R-HybridSN can better learn deep hierarchical spatial–spectral features and obtained excellent classification effect using very few training samples.

Although R-HybridSN achieved far better classification performance than all the contrast models, fixed window size and principle component number may not be the best choice for different hyperspectral datasets with various spatial and spectral resolution. In the future, in order to better exploit spatial correlations for different datasets, deformable convolutional networks [63] may be a workable solution. And in order to better learn spectral features, other efficient and novel high dimensional data mining methods, such as subspace clustering, can be combined with our proposed model.

## 5. Conclusions

On account of the fact that it is difficult to obtain enough labeled samples in supervised hyperspectral classification, we present R-HybridSN from the perspective of network optimization. Based on 2D-3D-CNN, residual connections and depth-separable convolution, R-HybridSN can learn deep hierarchical spatial–spectral features for hyperspectral classification. Rigorous comparative experiments were conducted on three public available hyperspectral data sets: Indian Pines, Salinas, and the University of Pavia. Experimental results indicate that the proposed R-HybridSN can achieve far better classification effect than all the contrast models using very few training samples.

In our research, PCA is used in the spectral dimension and a 3D patch with fixed size is extracted from hyperspectral data. However, it may not be the best choice for different hyperspectral datasets with various spatial and spectral resolutions. In our future researches, the nature of hyperspectral data should be paid more attention. In addition, some novel, effective high dimensional data processing methods, such as subspace clustering, should be further explored in hyperspectral classification. Constructing the deep learning model in this paper is from the perspective of network optimization. Subsequent studies can combine the idea of this paper with encoding–decoding learning [52] or generative adversarial learning [64]. Building the semi-supervised deep learning model using R-HybridSN as the base module might achieve better hyperspectral classification effect. Since the “small sample” hyperspectral classification is quite an open area, we hope that the ideas behind R-HybridSN can be expanded and go further.

## Figures and Tables

**Table 1:** The output data dimension and convolution kernel size of each layer in the network.

Layer Name	Output Dimension	Convolution Kernel Size	Convolution Kernel Number
Input	15, 15, 16, 1		
Conv3D_1	15, 15, 16,12	(1, 1, 3), (3, 3, 3), (5, 5, 3)	4 + 4 + 4
Conv3D_2	13, 13, 14, 16	(3, 3, 3)	16
Conv3D_3	11, 11, 12, 32	(3, 3, 3)	32
Conv3D_4	11, 11, 12, 40	(1, 1, 3)	40
Conv3D_5	9, 9, 10, 48	(3, 3, 3)	48
Conv3D_6	7, 7, 8, 64	(3, 3, 3)	64
Reshape	7, 7, 512		
Separable2D_1	4, 4, 128	(4, 4, 4)	128
Separable2D_2	1, 1, 512	(4, 4, 4)	512
Flatten	512		
FC	96		
Output	number of land-use categories		

**Table 2:** The overall accuracy of different models in Indian Pines ^1^.

Model\Amount of Training Samples	20%	10%	8%	6%	4%	2%
2D-CNN	91.23 ± 0.21	83.86 ± 1.00	82.43 ± 0.62	78.59 ± 0.79	74.92 ± 0.98	67.13 ± 1.12
M3D-DCNN	90.03 ± 2.18	80.10 ± 4.56	78.04 ± 2.13	74.57 ± 2.63	70.48 ± 3.21	62.28 ± 3.18
HybridSN	99.30 ± 0.18	97.66 ± 0.23	96.37 ± 1.19	93.64 ± 4.40	91.88 ± 1.39	83.14 ± 1.60
Model A	99.21 ± 0.24	96.94 ± 0.59	97.03 ± 0.43	93.80 ± 0.97	91.20 ± 1.61	81.97 ± 2.28
Model B	99.12 ± 0.20	96.47 ± 0.59	95.09 ± 0.52	91.46 ± 1.40	87.25 ± 1.07	73.81 ± 3.33
R-HybridSN	99.52 ± 0.16	98.44 ± 0.44	98.12 ± 0.35	96.69 ± 0.70	94.99 ± 0.39	86.67 ± 1.02

^1^ The standard deviation is shown after ±.

**Table 3:** The overall accuracy of different models in Salinas.

Model\Amount of Training Samples	5%	2%	1.6%	1.2%	0.8%	0.4%
2D-CNN	96.63 ± 0.24	94.67 ± 0.15	94.47 ± 0.24	93.82 ± 0.21	93.03 ± 0.26	91.38 ± 0.44
M3D-DCNN	92.65 ± 0.49	90.17 ± 0.56	88.68 ± 1.80	87.60 ± 1.35	86.82 ± 1.18	83.42 ± 1.60
HybridSN	99.83 ± 0.10	99.57 ± 0.25	99.53 ± 0.19	99.12 ± 0.33	97.78 ± 0.78	94.88 ± 0.90
Model A	99.62 ± 0.15	99.12 ± 0.19	98.64 ± 0.27	98.17 ± 0.41	96.73 ± 0.53	92.72 ± 0.94
Model B	99.46 ± 0.19	98.38 ± 0.33	97.51 ± 0.70	96.46 ± 1.04	93.74 ± 2.07	88.25 ± 1.93
R-HybridSN	99.82 ± 0.04	99.36 ± 0.14	99.18 ± 0.24	98.49 ± 0.60	96.97 ± 0.57	94.33 ± 0.48

**Table 4:** The overall accuracy of different models in University of Pavia.

Model\Amount of Training Samples	5%	2%	1.6%	1.2%	0.8%	0.4%
2D-CNN	96.59 ± 0.21	94.50 ± 0.40	93.55 ± 0.22	91.82 ± 0.56	89.98 ± 0.38	85.27 ± 0.90
M3D-DCNN	92.80 ± 0.95	89.27 ± 1.35	88.28 ± 1.47	87.19 ± 1.71	82.75 ± 2.84	76.53 ± 3.94
HybridSN	99.45 ± 0.09	97.86 ± 0.56	96.87 ± 0.31	95.86 ± 0.93	93.30 ± 1.41	85.95 ± 1.58
Model A	99.37 ± 0.12	97.99 ± 0.53	97.83 ± 0.64	95.01 ± 1.44	93.98 ± 1.70	90.85 ± 1.51
Model B	99.03 ± 0.10	96.89 ± 0.59	96.45 ± 1.02	88.54 ± 4.45	91.05 ± 2.33	81.06 ± 3.21
R-HybridSN	99.47 ± 0.14	98.47 ± 0.27	98.30 ± 0.21	96.40 ± 1.66	95.64 ± 0.52	91.60 ± 1.12

**Table 5:** Training time(s) of different models in Indian Pines.

Model\Amount of Training Samples	20%	10%	8%	6%	4%	2%
2D-CNN	14.6	8.9	7.4	6.6	5.0	4.0
M3D-DCNN	314.8	157.2	126.3	95.2	64.2	34.5
HybridSN	425.9	237.0	199.5	165.2	118.3	77.4
Model A	119.6	72.3	64.7	52.1	44.5	35.3
Model B	66.2	41.7	39.0	25.8	20.0	13.7
R-HybridSN	109.9	63.4	60.9	46.9	31.5	21.8

**Table 6:** Training and testing sample numbers in Indian Pines.

No.	Class Name	Total Labeled Samples	Training	Testing
1	Alfalfa	46	2	44
2	Corn-notill	1428	71	1357
3	Corn-mintill	830	41	789
4	Corn	237	12	225
5	Grass-pasture	483	24	459
6	Grass-trees	730	37	693
7	Grass-pasture-mowed	28	1	27
8	Hay-windrowed	478	24	454
9	Oats	20	1	19
10	Soybean-notill	972	49	923
11	Soybean-mintill	2455	123	2332
12	Soybean-clean	593	30	563
13	Wheat	205	10	195
14	Woods	1265	63	1202
15	Buildings-Grass-trees-drives	386	19	367
16	Stone-steel-towers	93	5	88
Total		10,249	512	9737

**Table 7:** Training and testing sample numbers in Salinas.

No.	Class Name	Total Labeled Samples	Training	Testing
1	Brocoli_green_weeds_1	2009	20	1989
2	Brocoli_green_weeds_2	3726	37	3689
3	Fallow	1976	20	1956
4	Fallow_rough_plow	1394	14	1380
5	Fallow_smooth	2678	27	2651
6	Stubble	3959	39	3920
7	Celery	3579	36	3543
8	Grapes_untrained	11,271	113	11,158
9	Soil_vinyard_develop	6203	62	6141
10	Corn_senesced_green_weeds	3278	33	3245
11	Lettuce_romaine_4wk	1068	11	1057
12	Lettuce_romaine_5wk	1927	19	1908
13	Lettuce_romaine_6wk	916	9	907
14	Lettuce_romaine_7wk	1070	11	1059
15	Vinyard_untrained	7268	72	7196
16	Vinyard_vertical_trellis	1807	18	1789
Total		54,129	541	53,588

**Table 8:** Training and testing sample numbers in University of Pavia.

No.	Class Name	Total Labeled Samples	Training	Testing
1	Asphalt	6631	66	6565
2	Meadows	18,649	186	18,463
3	Gravel	2099	21	2078
4	Trees	3064	31	3033
5	Painted metal sheets	1345	13	1332
6	Bare Soil	5029	50	4979
7	Bitumen	1330	13	1317
8	Self-Blocking Bricks	3682	37	3645
9	Shadows	947	10	937
Total		42,776	427	42,349

**Table 9:** Classification result of Lettuce_romaine_6wk by R-HybridSN ^2^.

	1	2	3	4	5	6	7	8	9	10	11	12	13	14	15	16
1	0	1	0	0	0	0	0	0	0	0	0	0	849	57	0	0
2	0	0	0	0	0	0	0	0	0	2	0	0	728	177	0	0
3	0	0	0	0	0	0	0	0	0	0	0	56	689	162	0	0
4	0	0	0	0	0	0	0	0	0	0	0	91	779	37	0	0
5	0	0	0	0	0	0	0	0	0	0	0	0	872	35	0	0
6	0	0	0	0	0	0	0	0	0	0	0	4	700	203	0	0
7	0	0	0	0	0	0	0	0	0	0	0	256	573	78	0	0
8	0	0	0	0	0	0	0	0	0	4	2	0	857	44	0	0
9	0	0	0	0	0	0	0	0	0	0	0	176	717	14	0	0
10	0	0	0	0	0	0	0	0	0	0	0	4	748	155	0	0

^2^ The classified land-use type index of the thirteenth class of ground object, Lettuce_romaine_6wk, in ten consecutive experiments.

**Table 10:** Classification results of different models in Indian Pines.

	2D-CNN	M3D-DCNN	HybridSN	Model A	Model B	R-HybridSN
1	7.95	27.50	61.82	30.91	29.55	45.00
2	70.69	59.15	92.25	91.16	88.81	95.45
3	52.84	45.07	92.97	90.08	85.17	97.36
4	27.51	38.49	78.22	76.27	67.20	94.80
5	90.44	70.33	96.60	97.58	94.44	98.85
6	98.59	97.20	98.11	99.06	97.65	99.32
7	10.37	18.52	68.52	62.22	37.41	95.56
8	99.96	98.04	99.96	99.85	99.47	100.00
9	16.32	25.79	83.68	61.58	11.05	65.26
10	67.84	55.85	96.12	93.07	86.16	95.90
11	78.16	76.20	96.66	95.53	93.31	98.09
12	42.01	33.89	85.44	83.46	79.01	89.15
13	98.97	91.23	94.97	98.41	96.92	99.74
14	97.65	94.68	99.34	98.51	98.05	99.26
15	62.62	42.37	82.92	80.52	74.01	87.66
16	76.02	49.32	80.00	85.11	83.18	88.18
Kappa	0.718 ± 0.010	0.642 ± 0.045	0.934 ± 0.012	0.920 ± 0.008	0.883 ± 0.012	0.960 ± 0.004
OA(%)	75.47 ± 0.81	68.88 ± 3.77	94.24 ± 1.01	92.97 ± 0.73	89.80 ± 1.03	96.46 ± 0.33
AA(%)	62.37 ± 1.64	57.73 ± 6.52	87.97 ± 1.93	83.96 ± 2.41	76.34 ± 2.36	90.60 ± 1.53

**Table 11:** Classification results of different models in Salinas.

	2D-CNN	M3D-DCNN	HybridSN	Model A	Model B	R-HybridSN
1	99.97	94.88	99.99	100	99.94	100.00
2	99.86	99.61	100	99.82	99.85	99.97
3	99.43	91.89	99.82	99.32	98.32	99.49
4	98.83	98.33	98.38	99.26	98.09	98.72
5	96.77	98.83	99.26	98.17	98.31	98.43
6	99.79	98.09	99.93	99.80	99.88	99.90
7	99.33	97.67	99.95	99.94	98.88	99.96
8	87.39	82.40	97.77	97.84	92.43	98.23
9	99.97	98.14	99.99	99.51	99.79	99.99
10	93.98	87.60	98.36	98.04	95.45	97.90
11	89.62	86.72	96.06	95.87	90.40	96.46
12	99.99	96.99	97.44	98.12	95.67	99.09
13	98.52	97.14	97.42	77.46	74.10	82.82
14	97.64	91.78	99.52	93.39	92.72	97.25
15	79.46	64.42	97.06	89.79	86.69	95.12
16	95.71	78.14	100	99.16	98.56	99.71
Kappa	0.928 ± 0.003	0.867 ± 0.002	0.985 ± 0.007	0.968 ± 0.010	0.945 ± 0.012	0.980 ± 0.004
OA(%)	93.55 ± 0.26	88.02 ± 1.35	98.72 ± 0.59	97.15 ± 0.90	95.07 ± 1.08	98.25 ± 0.40
AA(%)	96.02 ± 0.42	91.41 ± 0.81	98.81 ± 0.5	96.59 ± 2.42	94.94 ± 2.12	97.69 ± 0.69

**Table 12:** Classification results of different models in University of Pavia.

	2D-CNN	M3D-DCNN	HybridSN	Model A	Model B	R-HybridSN
1	96.88	90.56	95.72	95.40	96.53	96.94
2	99.01	89.47	99.68	99.36	99.00	99.69
3	75.08	59.11	84.38	87.60	78.59	87.17
4	87.74	93.25	87.70	89.16	87.71	89.15
5	98.17	93.66	98.99	99.50	98.86	99.51
6	75.51	69.63	96.82	96.59	94.68	98.44
7	61.32	65.71	84.42	94.37	94.66	95.82
8	80.61	78.35	89.18	88.08	84.25	93.28
9	97.97	94.41	71.71	79.51	81.28	77.82
Kappa	0.881 ± 0.008	0.798 ± 0.016	0.935 ± 0.011	0.941 ± 0.013	0.927 ± 0.097	0.955 ± 0.007
OA(%)	91.13 ± 0.55	84.63 ± 1.21	95.09 ± 0.80	95.55 ± 0.99	94.50 ± 0.72	96.59 ± 0.50
AA(%)	85.81 ± 1.48	81.57 ± 1.79	89.84 ± 1.93	92.17 ± 1.91	90.62 ± 1.79	93.09 ± 1.20

**Table 13:** The comparison between whether the third residual connection is used in R-HybridSN ^3^.

	Indian Pines		Salinas		University of Pavia	
Kappa	0.960 ± 0.004	0.927 ± 0.012	0.980 ± 0.004	0.975 ± 0.005	0.955 ± 0.007	0.953 ± 0.006
OA	96.46 ± 0.33	93.63 ± 0.011	98.25 ± 0.40	97.78 ± 0.41	96.59 ± 0.50	96.45 ± 0.43
AA	90.60 ± 1.53	85.28 ± 0.029	97.69 ± 0.69	97.75 ± 0.72	93.09 ± 1.20	94.15 ± 0.86

^3^ The classification results of R-HybridSN and R-HybridSN without the third residual connection are shown in the left and right side below every dataset, respectively.
